# Temporal integration of infrasound at threshold

**DOI:** 10.1371/journal.pone.0289216

**Published:** 2023-07-31

**Authors:** Björn Friedrich, Holger Joost, Thomas Fedtke, Jesko L. Verhey

**Affiliations:** 1 Department of Experimental Audiology, Otto von Guericke University Magdeburg, Magdeburg, Germany; 2 Physikalisch-Technische Bundesanstalt, Braunschweig, Germany; University of Miami, UNITED STATES

## Abstract

Infrasounds are signals with frequencies below the classical audio-frequency range, i.e., below 20 Hz. Several previous studies have shown that infrasound is audible as well, provided that the sound level is high enough. Hence, the sound pressure levels at threshold are much higher than those in the classical audio-frequency range. The present study investigates how the duration and the shape of the temporal envelope affect thresholds of infrasound stimuli in quiet. Two envelope types were considered: one where the duration of the steady state was varied (plateau bursts) and one where the number of consecutive onset–offset bursts was varied (multiple bursts). Stimuli were presented monaurally to human listeners by means of a low-distortion sound reproduction system. For both envelope types, thresholds decrease with increasing duration, a phenomenon often referred to as temporal integration. At the same duration, thresholds for plateau-burst stimuli are typically lower than those for multiple-burst stimuli. The data are well described by a slightly modified version of a model that was previously developed to account for temporal integration in the classical audio-frequency range. The results suggest similar mechanisms underlying the detection of stimuli with frequencies in the infrasound and in the classical audio-frequency range. Since the model accounts for the effect of duration and, more generally, the shape of the envelope, it can be used to enhance the comparability of existing and future datasets of thresholds for infrasounds with different temporal stimulus parameters.

## Introduction

Several sources in our environment emit infrasound, i.e., sound with frequencies below 20 Hz [1, 2]. These include both natural sources, e.g., microbaroms [3], thunderstorms [4], earthquakes, and volcanic activity, as well as anthropogenic sources, e.g., rocket launches [5], military and industrial blasts, mining activities, geothermal plants, heat pumps, and wind turbines [6, 7]. An increase in exposure of humans to infrasound is to be expected in several countries as the transition towards renewable energy resources advances. Also, the number of natural sources may increase due to climate-change related severe-weather phenomena. Several studies have examined the effects of infrasound on health and well-being (see, e.g., the reviews by Carlile, Davy, Hillman, and Burgemeister or by Baliatsas, van Kamp, van Poll, and Yzermans [8, 9]). Yet even the basic mechanisms underlying infrasound perception are still not well understood.

Studies on human brain activation show that the brain responds to infrasound presented to the ear [10–14]. The latter three studies reveal an activation of the primary auditory cortex, which supports the hypothesis that infrasound is processed by the auditory system. In agreement with this hypothesis, several psychoacoustic studies have shown that pure tones with frequencies down to at least 2 Hz can be detected by the auditory system (e.g., [15–20]; for a review, see Møller and Pedersen [21]). The sound pressure levels at threshold—in the following simply referred to as *thresholds—*are higher than those for frequencies in the classical audio-frequency range (between 20 Hz and 20 kHz) and increase with decreasing frequency.

Thresholds do not only depend on frequency but also on the envelope and, thus, also on the duration of the stimuli. Several studies on the perception of sounds with frequencies in the classical audio-frequency range show that thresholds tend to decrease with increasing stimulus duration, an effect which is commonly referred to as *temporal integration* (e.g., [22–25]). For stimuli with frequencies between 500 Hz and 4 kHz, thresholds decrease by about 20/2 dB to 20/3 dB per tenfold increase in stimulus duration, equivalent to about 3 dB to 2 dB per doubling of the duration [25]. Recent data from Jurado, Larrea, Patel, and Marquardt [20], based on measurements in five ears of five normal-hearing listeners, suggest that temporal integration also occurs in the low-frequency (32 Hz) and infrasound (16 Hz and 4 Hz) ranges, although the decrease in threshold seems to be smaller (about 20/4 dB per tenfold increase in stimulus duration). This smaller decrease could be due to the long stimulus durations (> 1 s) that were considered in their study, which are considerably longer than durations commonly used in studies on temporal integration in the classical audio-frequency range.

Several studies argue that temporal integration may be modelled by a leaky integrator with a time constant of about 100 ms to 200 ms (see, e.g., [26], for an overview). Such a model predicts that thresholds would hardly change at long durations. Fastl and Zwicker argued that this is already the case for stimuli that are longer than 200 ms (Fig 4.18 in [27]; however, see [25]). An upper limit of 200 ms was also reported for loudness [28]. It is unlikely that a 200 ms limit applies to infrasound. Such a limit would cover less than a full cycle for frequencies lower than 5 Hz. Also, data sets of temporal integration under masking indicate that the time constant presumed in leaky-integrator models decreases as the frequency of the tone increases [29, 30]. Thus, if temporal-integration data for frequencies in the infrasound range were modeled with a leaky integrator, the time constant estimated would presumably be much longer than for frequencies in the classical audio-frequency range.

Some authors assume that, at least for infrasound, the more important temporal stimulus parameter is the number of cycles with respect to the given signal frequency and not the duration in milliseconds. For example, Jurado, Larrea, Patel, and Marquardt [20] used tone bursts that were switched on and off by ramps with a duration of one cycle each. Kühler, Fedtke, and Hensel [17] used infrasound stimuli that included at least three cycles per ramp and four cycles for the plateau (i.e., the part of the stimulus with a constant amplitude between the ramps). As a consequence, the durations of such infrasound stimuli can be considerably longer than the durations commonly used in the classical audio-frequency range. Moreover, if the number of cycles was the key factor of temporal integration, the temporal-integration function would depend on the signal frequency. The importance of the number of cycles for the temporal integration of infrasound stimuli has not been examined before and will be addressed in the present study. In addition, it will be investigated whether threshold–duration functions saturate as suggested by, e.g., a leaky-integrator model, for durations considerably longer than the time constant of the leaky integrator.

Apart from the duration, other stimulus attributes influence the shape of the temporal-integration function. For example, Gerken, Bhat, and Hutchison-Clutter [24] as well as Heil, Matysiak, and Neubauer [25] showed that detection thresholds of equally long stimuli with a plateau in the envelope (plateau burst, PB) are lower than those for a sequence of fully flanked stimuli (multiple bursts, MB). For an MB stimulus, the duration of the gaps between individual bursts has also been shown to affect thresholds [24, 25, 31–34]. It is likely that the shape of the envelope also affects the detection of infrasound stimuli, but this has not yet been confirmed experimentally. One important aspect of the envelope are the ramps at stimulus on- and offset. Their durations are often long for infrasound stimuli and, thus, may have a considerable impact on the temporal integration, but this has not yet been investigated. This study aims to fill these gaps.

In this study, experimental data on temporal integration of stimuli with carrier frequencies in the infrasound range (8 Hz and 16 Hz) and with two types of envelopes (MB and PB) are presented. The MB envelope type was chosen because it allows examining how threshold changes with the number of equal stimulus portions. For 16 Hz, two different ramp durations were used to examine the effect of this stimulus parameter on threshold. Detection thresholds in quiet were measured over a range of stimulus durations from 375 ms to 3000 ms. The data are compared to the prediction of a slightly modified version of a physiologically motivated probabilistic model of temporal integration by Heil, Matysiak, and Neubauer [25] that was originally developed to account for temporal integration data for tonal stimuli in the classical audio-frequency range. The data are also compared to existing datasets of infrasound thresholds from the literature. To compare the results of the present study to the data of Jurado, Larrea, Patel, and Marquardt [20], the model is used to account for differences in the stimulus envelopes.

## Methods

### Listeners

Twelve human listeners (seven female, five male) with ages between 20 and 38 years (median: 25 years) participated in the experiment. Nine were paid volunteers and three were members of the Department of Experimental Audiology of the Otto von Guericke University Magdeburg. All listeners were otologically normal as confirmed by a questionnaire for hearing testing (Annex A of ISO 389–9 [35]). Audiometric hearing levels of those ears that were used for the infrasound experiment were lower than or equal to 15 dB for all audiometric frequencies between 125 Hz and 2 kHz. Listeners were instructed about the goal of the study and about the pseudonymized use of their data according to the General Data Protection Regulation (GDPR) of the European Union. The Declaration of Helsinki was adhered to in all measurements. Ethics approval was obtained from the ethics committee of the medical faculty of the Otto von Guericke University Magdeburg (ethics approval 79/17).

### Stimuli and experimental conditions

Stimuli were sinusoids with a frequency of 8 Hz or 16 Hz. Two types of stimulus envelopes were used: plateau bursts (PB) and multiple bursts (MB). To implement different stimulus durations, envelopes were constructed based on equally long segments with specific properties: onset and offset segments were rising and falling raised-cosine ramps, respectively. The plateau was subdivided into segments with the same duration as the onset or offset segment. These plateau segments had a constant amplitude. Based on the specific combination of the segments, stimuli were labelled as follows: PB*m* consisted of one onset segment (i.e., the rising ramp at stimulus onset), one offset segment (i.e., the falling ramp at stimulus offset), and *m* plateau segments in between; MB*m* consisted of one onset segment followed by one offset segment, lined up *m* times without silent gaps between the bursts.

**Fig 1:**
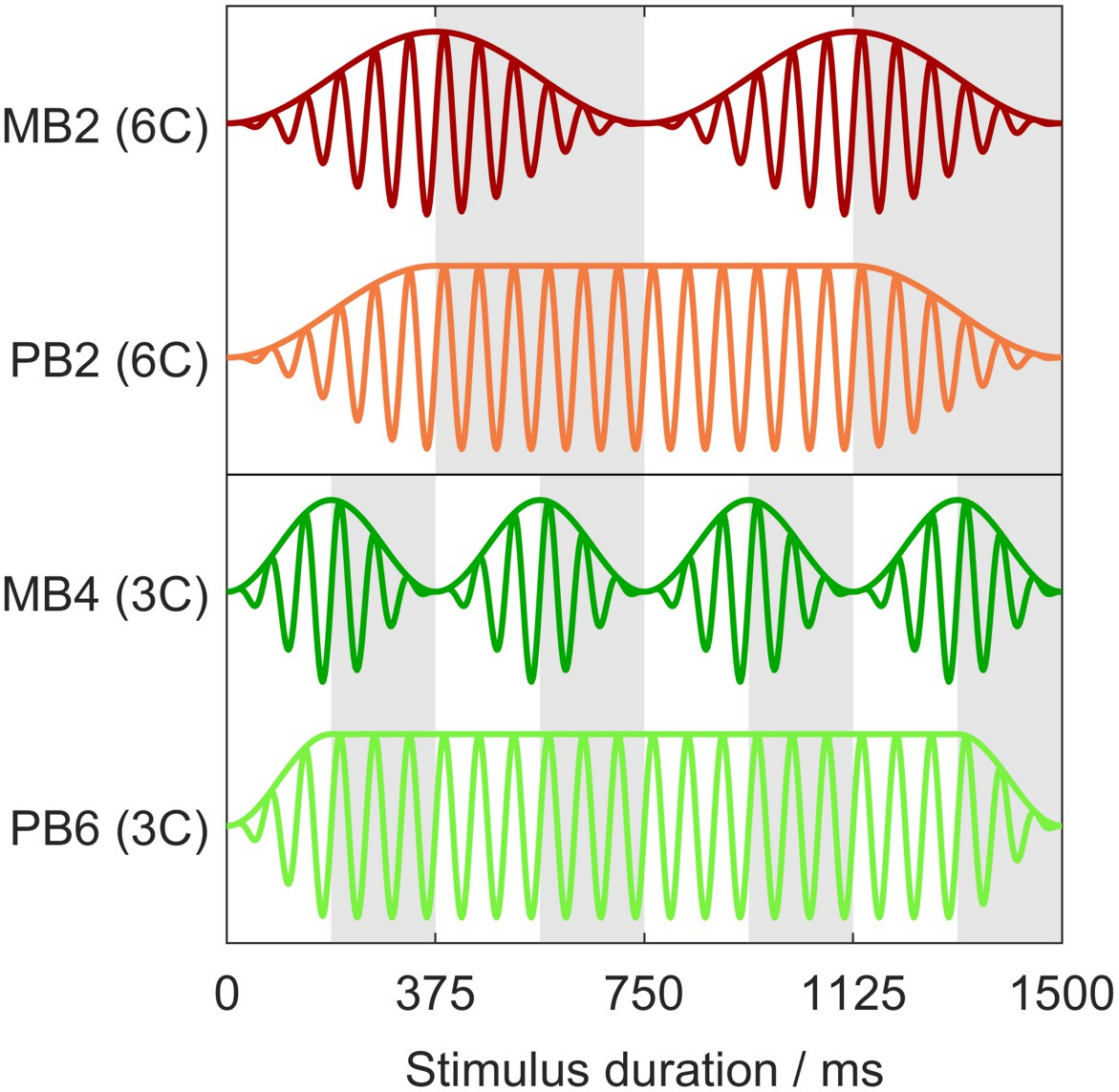
Examples of stimuli used in the experiment. Colored solid lines show time signals and envelopes of four stimuli with a carrier frequency of 16 Hz and a stimulus duration of 1500 ms. Alternating white–gray patterns indicate the segmentation of the stimuli.

Each segment had a duration *T*_B_ of either three cycles (3C) or six cycles (6C) of the carrier frequency. The experiment comprised three conditions, which are labelled according to the carrier frequency and the number of cycles per segment: 8Hz_3C, 16Hz_6C, and 16Hz_3C. Thus, *T*_B_ was either 187.5 ms (16Hz_3C) or 375 ms (8Hz_3C and 16Hz_6C). The total duration of a PB*m* stimulus was (2 + *m*) ·*T*_B_ and that of an MB*m* stimulus 2*m* · *T*_B_. Seven envelopes were used: PB0 (which is identical to MB1), PB1, PB2, PB6, MB2, MB3, MB4. Combining them with the three conditions resulted in 21 distinct stimuli that were used in the experiment. Their durations in milliseconds and total numbers of cycles are summarized in Table 1. Examples of the stimuli are shown in Fig 1.

**Table 1:** Stimulus durations in milliseconds and total numbers of cycles for each of the seven envelopes (rows) in the three experimental conditions (columns).

Envelope	8Hz_3C		16Hz_6C		16Hz_3C	
	Duration in ms	Cycles	Duration in ms	Cycles	Duration in ms	Cycles
PB6	3000	24	3000	48	1500	24
PB2	1500	12	1500	24	750	12
PB1	1125	9	1125	18	562.5	9
PB0 = MB1	750	6	750	12	375	6
MB2	1500	12	1500	24	750	12
MB3	2250	18	2250	36	1125	18
MB4	3000	24	3000	48	1500	24

### Measurement setup and procedure

All stimuli were generated digitally in MATLAB (MathWorks, Natick, MA) with a sampling rate of 96 kHz and a resolution of 24 bit. The resulting signals were converted from digital to analog via an external sound card (RME Fireface, Haimhausen, Germany) and finally presented monaurally to the ear with a custom-made low-distortion sound reproduction system (LDREPS) [36]. A key part of the LDREPS is the audiometric earphone transducer RadioEar DD45 mounted in an air-sealed aluminum housing with a sound outlet in the front plate. A sound tube with a length of 25 cm connects the sound outlet to the ear insert of an Etymotic ER-10B+ low-noise microphone system. Joost, Friedrich, Verhey, and Fedtke [18] showed that stimuli with frequencies of 8 Hz and 16 Hz were reproduced by the LDREPS with low distortions.

The LDREPS used in the present study is a slightly modified version of that used in [18]. For safety reasons, the LDREPS used here incorporates, instead of a low-pass filter, a rapid frequency-dependent level-watching protective switch in the signal path between amplifier and transducer. The switch interrupts the signal as soon as the signal level exceeds a given frequency-dependent maximum value, i.e., the 80 phon equal-loudness level contour [37]. The ear insert of the LDREPS was fitted to the right ear (eleven listeners) or the left ear (one listener). The second channel of the LDREPS was not used in the present experiment.

To ensure the proper fit of the ear insert, the sound pressure level of a 4 Hz signal, which had been calibrated in a B&K 4157 occluded-ear simulator (Brüel & Kjær, Nærum, Denmark), was always measured in situ by means of the inbuilt low-noise microphone of the LDREPS prior to the next run of an experimental condition.

During the experiment, listeners were seated comfortably in a double-walled sound-insulated booth. To ensure that the (non-expert) listeners were familiar with the infrasound stimuli, listeners were presented with a set of examples for the MB and PB stimuli at the beginning of the first session. Listeners were allowed to adjust the level by means of a slider (max. 60 phon) and to replay the examples as often as they wanted. If requested by the listeners, examples were also presented at the beginning of each experimental run.

Detection thresholds were measured using a three-interval, three-alternative forced-choice (AFC) procedure. The intervals of a trial were separated by 225 ms of silence. The intervals of a trial were highlighted on a screen, which was positioned in front of the listener. A randomly chosen interval contained the target stimulus. The listener was requested to press a numbered button on a hand-held keyboard to indicate which of the intervals contained the target stimulus. The levels of the target for the next trial were chosen on the basis of the listeners response with a one-up two-down rule, which converges to the level for which the probability of a correct response is $p_{corr}=1/\surd 2\approx 70.71\%$ [38, 39]. The adaptive procedure started at a sound pressure level that roughly corresponded to a loudness level of 20 phon (derived from [21]). The maximum sound pressure level corresponded to a loudness level of 40 phon (derived from [21]). The initial step size was 4 dB; it was halved after each upper reversal until a final step size of 1 dB was reached. The run continued with the final step size for four reversals. The arithmetic mean of the sound pressure levels at these four reversals was taken as the threshold estimate of the corresponding run. For each stimulus, thresholds were measured three times (one threshold per run). The arithmetic mean of the three threshold estimates was taken as the final threshold estimate of the corresponding stimulus.

### Model of temporal integration

In the present study, a modified version of the physiologically inspired probabilistic model of temporal integration proposed by Heil, Matysiak, and Neubauer [25] was used. The structure and the behavior of the model are described in detail in S1 File (Model description). Here, just the most important aspects are outlined.

The auditory stimuli are represented in the model by so-called “event” rates, which can be thought of as generalized neuronal firing rates. These are modelled by an inhomogeneous Poisson process, which is often assumed to describe neuronal firing rates (e.g., [40]). The original model has three main parameters: a spontaneous rate *λ*_spont_, an exponent *α*, and a gain factor *g*. Because *g* and *α* interact, undesirable numerical effects may occur when fitting this model to the high detection thresholds of infrasound stimuli, which may well exceed sound pressure levels of 100 dB. Therefore, instead of the gain factor of the original model, an attenuation *A* was used, which is related to *g* via the following relationship:

$$A=-\frac{20}{\alpha }\cdot \text{lg}\left(g\right)dB.$$ (1)

Another difference is that the original model described in [25] used filtered stimulus envelopes, whereas the model in the present study does not include a peripheral filter, since the properties of such a hypothetical filter for infrasound stimuli are unclear.

With these modifications, the condition for the detection of a stimulus with maximum amplitude *M* and normalized, time-dependent envelope *f*(*t*) can be expressed as:

∫0dλtdt≥ncrit,withλt=λspont+10-α⋅A20dB⋅M⋅ftα. (2)

Here, *d* denotes the stimulus duration over which the event rates are integrated, and *n*_crit_ is the critical number of events that is required for the stimulus to be detected. This critical number of events depends on the number of spontaneous events, *n*_spont_ = *λ*_spont_·*d* as well as on the specificities of the psychophysical procedure [25, 41, 42]. The detection threshold, expressed in terms of decibels, is given by:

$$L=20\cdot \text{lg}\left(M\right)dB=A+\frac{20}{\alpha }\cdot \text{lg}\left(\frac{n_{crit}-n_{spont}}{\int_{0}^{d}f^{\alpha }\left(t\right)dt}\right)dB.$$ (3)

To understand how changes in the parameters *α*, *A*, and *λ*_spont_ of the model affect the predicted threshold–duration functions, simulations were conducted in which one parameter was systematically varied while the other two were held constant. It was additionally investigated, how various ramp durations of MB and PB stimuli affect the predicted thresholds. Details of these simulations can be found in the section *Model behavior* in S1 File description. The main insights are:

(1) An increase in the exponent *α* leads to a decrease in the slope of the threshold–duration function. The difference between the threshold–duration functions for different values of the exponent is largest when stimuli are very short or very long.
(2) Changing the attenuation *A* results in a vertical shift of the threshold–duration functions. The vertical distance of two threshold–duration functions with different values for *A* is equal to the difference between the two *A* values. This further motivates the choice of the parameter *A*, since it is easier to interpret than the gain factor *g* of the original model.
(3) When the spontaneous rate *λ*_spont_ is zero, the model predicts, for the MB stimuli, a linear relation between threshold and the logarithm of the number of bursts *m* and, hence, between threshold and the logarithm of stimulus duration *d* (cf. section 3 in S1 File description). A non-zero spontaneous rate, however, introduces a curvature into the threshold–duration functions, mostly affecting thresholds at the longest stimulus durations. This curvature is best seen when spontaneous rates are low. At higher spontaneous rates, threshold–duration functions appear to be shifted as a whole.
(4) An increase in ramp duration has distinct effects for MB and PB stimuli. For MB stimuli, it leads to a decrease in the differences (in threshold) between consecutive functions. For PB stimuli, it leads to an increase in the slope of the functions, and all functions converge towards longer stimulus durations.

## Results

### Experimental data

**Fig 2:**
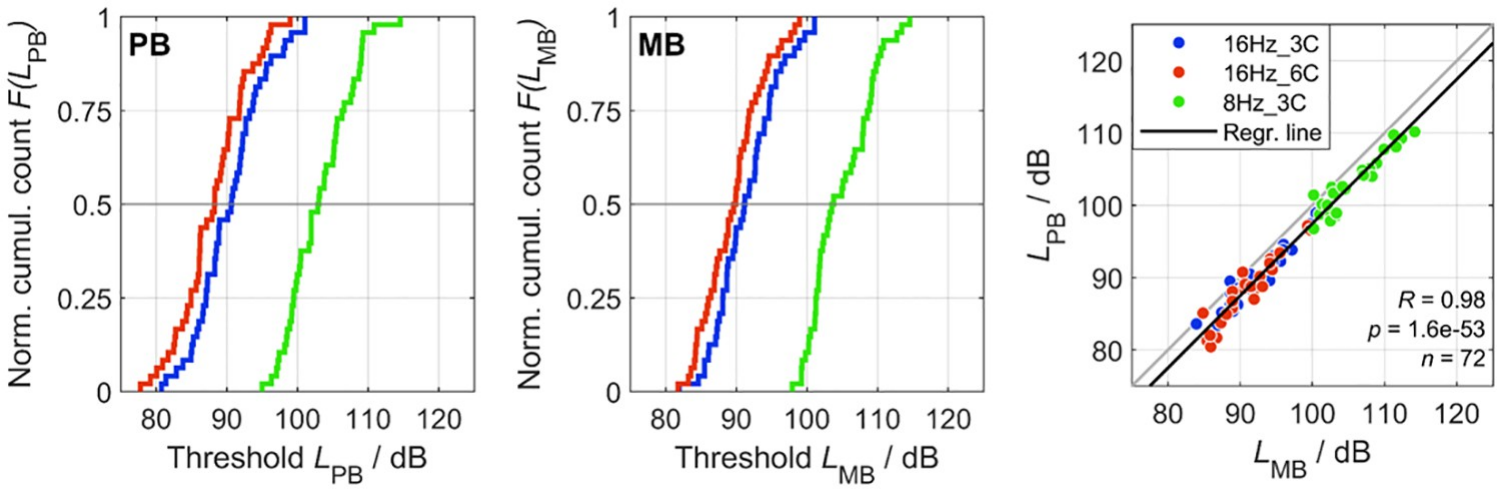
Individual thresholds of MB and PB stimuli. The left and the middle panel show the cumulative distribution functions of individual thresholds of MB and PB stimuli, respectively. The colors blue, red, and green identify different conditions. The right panel shows the Deming regression on pairs of thresholds of MB and PB stimuli with the same duration and of the same condition. The identical thresholds of MB1 and PB0 were excluded. The regression line (black) is located below the diagonal line (gray). The thresholds are strongly correlated. See text for details.

The left and middle panel of Fig 2 show empirical cumulative distributions of the individual thresholds of MB and PB stimuli, respectively. For each condition, the cumulative counts, *F*(*L*), are normalized with respect to the number of observed thresholds (12 listeners × 4 envelopes, i.e., *n* = 48 per condition). Thresholds vary over a range from 80.4 dB to 115.5 dB, with the lowest thresholds measured in the 16Hz_6C condition and the highest in the 8Hz_3C condition. An effect of condition on thresholds is also reflected in the central values of the distributions, i.e., the levels *L*_0.5_, for which fifty percent of all thresholds of a specific envelope type (MB or PB) are below this level (crossing points between dotted line at *F*(*L*_0.5_) and the cumulative distributions in the left and middle panel of Fig 2). The central values are close to monaural and binaural infrasound thresholds reported in the literature [17–21], i.e., between about 86 dB and 90 dB for 16 Hz stimuli and between about 101 dB and 104 dB for 8 Hz stimuli.

The right panel of Fig 2 shows the results of a Deming regression (MATLAB implementation from [43]) performed on pairs of individual thresholds for MB and PB stimuli. Each data point shows the thresholds of an MB stimulus and a PB stimulus from the same condition and having the same duration. The three different conditions (8Hz_3C, 16Hz_3C, or 16Hz_6C) are shown with different colors using the same color code as in the other two panels of this figure. For the analysis, only the two MB–PB pairings MB2–PB2 and MB4–PB6 could be considered, as their envelopes had the same duration (see Table 1). A regression line was fitted to these 72 data points (twelve listeners × three conditions × two pairings). Thresholds for the two envelope types (MB and PB) are strongly correlated (*R* = 0.98, *p* = 1.6 · 10^−53^). For the range of thresholds of the present study, the regression line (black) is located slightly below the diagonal (gray line). This means that thresholds of PB stimuli are generally lower than thresholds of MB stimuli of the same durations. This agrees with the corresponding results in the classical audio-frequency range for these two envelope types [24, 25].

To decide which kind of statistic measure is appropriate to characterize the average thresholds across listeners and which function has to be used for the fitting of the model parameters, individual thresholds were tested for normal distributions. For each condition and for each envelope type, Kolmogorov-Smirnov tests were employed to test the null hypothesis that the z-scored individual thresholds follow a standard-normal distribution. The null hypothesis could not be rejected for any of the conditions and envelope types (all *p* > 0.171). Thus, arithmetic statistics were used to characterize the average and the spread of thresholds across listeners and the squared-error-loss function was used for fitting the model parameters.

**Fig 3:**
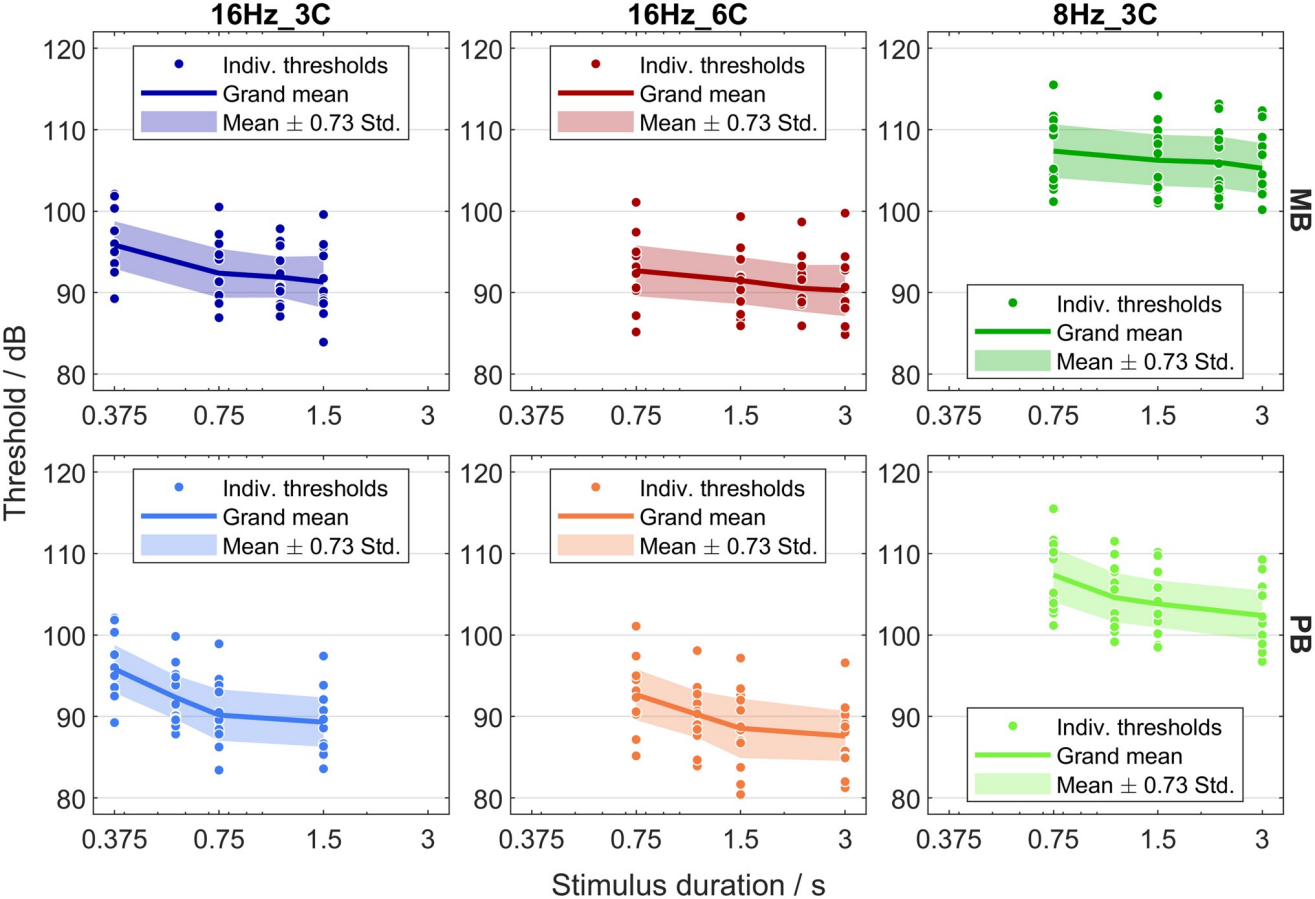
Thresholds as a function of stimulus duration. Each column and each of the colors blue, red, and green represents a different condition. Each row and each color shading (dark versus light) represents a different envelope type. Dots represent data of individual listeners. Solid lines represent grand means and shaded tubes represent ranges of the grand mean ± 0.73 times the standard deviation.

Fig 3 shows thresholds as a function of stimulus duration for the three conditions (columns) and the two envelope types (rows). Individual thresholds are shown as dots. Solid lines indicate the grand means, computed across listeners, and shaded regions the corresponding range of the grand mean ± 0.73 times the standard deviation. For a normal distribution, 50% of the data fall in this range. The factor 0.73 was chosen to allow for comparisons of these intervals with inter-quartile ranges that have been used in several previous studies (e.g., [17–19]). The range hardly varies with stimulus duration, i.e., the data are homoscedastic. Homoscedasticity is desirable when a squared-error-loss function is used for fitting the model to the data (e.g., [44]).

In all conditions, thresholds decrease with increasing stimulus duration, reflecting temporal integration at threshold. In general, the grand-mean threshold–duration functions for the MB stimuli (Fig 3, top row) decrease less with increasing duration than the corresponding threshold–duration functions for the PB stimuli (Fig 3, bottom row). These latter functions are more curved than those for the MB stimuli with a shallower slope at long durations than at short durations. Note that none of the threshold–duration functions seems to saturate in the range of durations considered here. These findings are in qualitative agreement with results reported for the classical audio-frequency range (e.g., Figs 3 and 4 in [25]).

### Model predictions

The lowest center frequency of an auditory filter is somewhere between 50 Hz and 80 Hz [45]. Thus, it is likely that the cochlea is unable to spectrally resolve frequencies in the infrasound range. It is, however, possible that infrasound is processed in the auditory filter with the lowest center frequency. The model simulations are based on this assumption, i.e., the 16 Hz and 8 Hz stimuli are processed within the same auditory filter, irrespective of the shape of the envelope. This assumption implies that the exponent as well as the spontaneous rate of the model should be the same *within* the auditory filter for the stimuli used in this study. The attenuation, in contrast, may depend on the frequency of the stimulus as it comprises all attenuations within the system, including the attenuations of outer and middle ear filtering.

Based on this assumption, the model was first fitted to the thresholds of the 16 Hz stimuli by varying the parameters *α*, *A*, and *λ*_spont_. In a second step, the model was fitted to the 8 Hz data by varying only the parameter *A* while *α* and *λ*_spont_ were set to those values that were obtained for 16 Hz. The results of the best fits are summarized in Table 2. The parameter values *α* = 2.7 and *λ*_spont_ = 0.26/s are close to those reported for the classical audio-frequency range (i.e., *α* = 3 and *λ*_spont_ = 0.58/s and 1.59/s in different experiments; [25]). The best fit to the 8 Hz data yielded an attenuation of *A* = 103.4 dB, which is about 15 dB higher than the attenuation for the 16 Hz data. The difference between prediction and data is given as RMS error; their values of 0.55 dB are small.

**Table 2:** Parameters of the best model fits to the thresholds of 8 Hz and 16 Hz stimuli.

Carrier frequency	Exponent *𝛂*	Spont. rate *λ*_spont_	Attenuation *A*	RMS error
16 Hz	2.7	0.26/s	88.6 dB	0.55 dB
8 Hz	2.7	0.26/s	103.4 dB	0.55 dB

**Fig 4:**
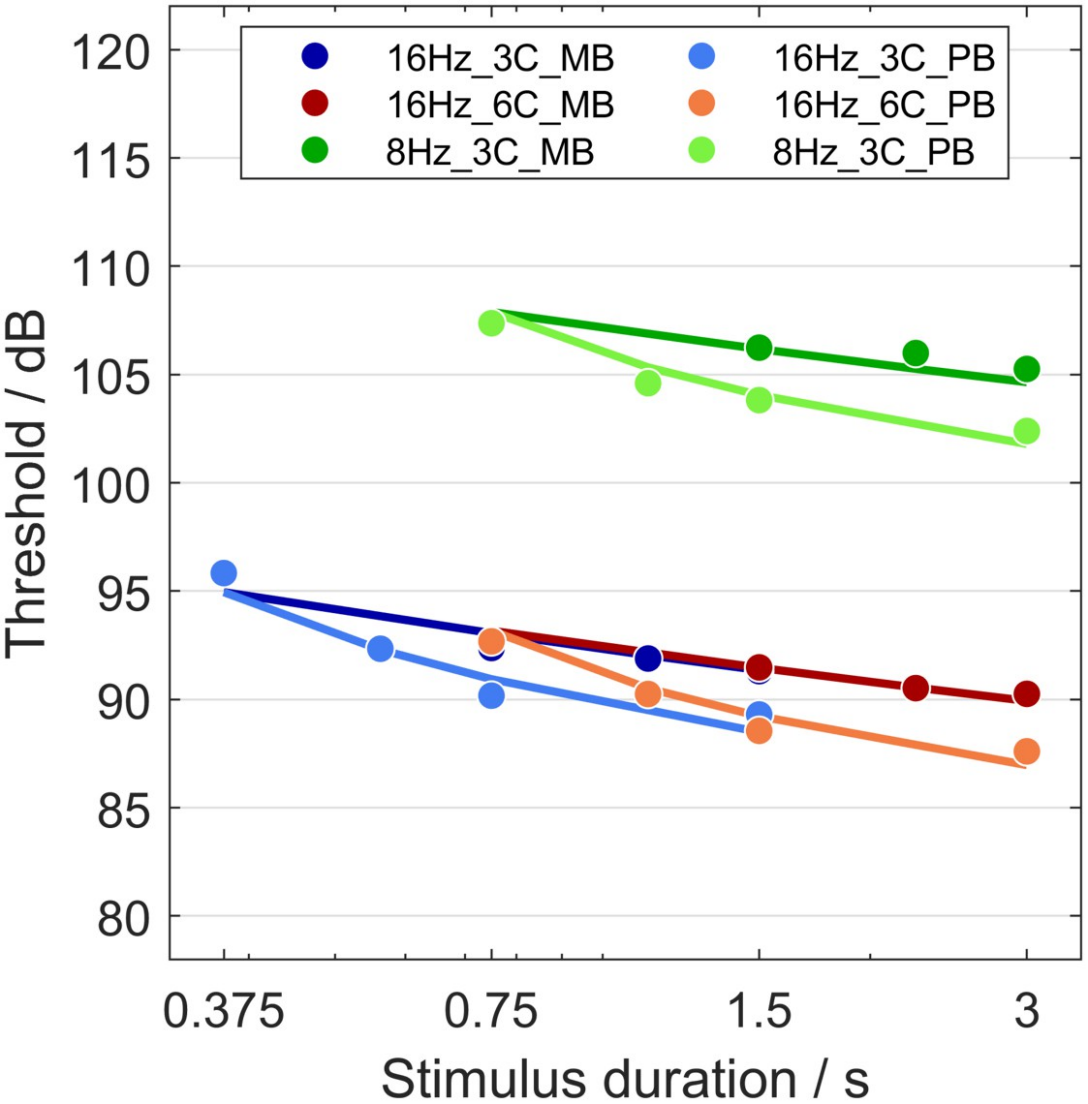
Fits of the model to the grand-mean thresholds of the MB and PB stimuli. Each color identifies a different condition, and color shading identifies the envelope type. Dots represent measured grand-mean thresholds and solid lines the corresponding threshold–duration functions predicted by the model. See text for detail.

Fig 4 shows the predicted threshold–duration functions (solid lines) with the model parameters of Table 2 together with the grand-mean data (dots). In all three conditions (8Hz_3C, 16Hz_6C, and 16Hz_3C), the predicted threshold–duration functions for the MB stimuli are almost straight lines, whereas the functions for the PB stimuli are more curved. The threshold–duration functions for the MB and the PB stimuli depart from each other as the stimulus duration increases. This agrees with results reported for the classical audio-frequency range (e.g., Figs 3 and 4 in [25]). The two predicted threshold–duration functions for the 16 Hz MB stimuli (16Hz_6C and 16Hz_3C) nearly overlap for the common durations of the two conditions.

The last data point of each curve is always higher than the predicted thresholds. Thus, it may be that temporal integration is slightly overestimated by the model. Note, however, that the RMS difference between measured and predicted thresholds across the six last points is only 0.6 dB, considerably smaller than the RMS of the inter-individual standard deviations that belong to the six last measured thresholds, i.e., 4.2 dB.

## Discussion

### Multiple bursts

The threshold–duration functions for the MB stimuli are shallower than those for the corresponding PB stimuli. This agrees with the data in the classical audio-frequency range [24, 25], indicating a similar mechanism for the processing of stimuli in the infrasound- and the classical audio-frequency range. A similar processing mechanism is supported also by the results of the modelling. The exponent of the model used to account for the infrasound data of the present study is close to 3, the integer exponent found to describe well a variety of temporal-integration data (e.g., [25, 32, 41]), spectral- and spectrotemporal integration data [42], and binaural integration data [46] for stimuli in the classical audio-frequency range. The spontaneous rate of the model is below 1/s. This agrees with the parameter values reported in [25] (i.e., *λ*_1_ = 1.59/s and *λ*_1_ = 0.58/s in their Fig 11) and in [32] (i.e., *λ*_1_ = 0.09/s in their Fig 3). The attenuation is considerably higher than those for the classical audio-frequency range (after converting the gain factor using Eq (1)), but this was expected for the considered frequencies (8 Hz and 16 Hz), because the thresholds for these frequencies are considerably higher than for the frequencies that were considered in the literature for the fitting of the model.

In section 3 of S1 File, a prediction of the threshold–duration function for MB stimuli is derived under the assumption that the spontaneous rate were 0/s. The resulting function (cf. equation (8) in S1 File) would be linear when plotted as a function of lg(*m*), where *m* is the number of bursts of an MB*m* stimulus. It follows that, under this assumption, the slope of this function could be directly used to estimate the exponent. An exponent of 3 would result in a slope *s*, expressed as threshold change per doubling of duration, of -2 dB. Table 3 shows the slopes, *s*, and the offsets, *L*_O_, of straight lines fitted to the grand mean thresholds of the MB stimuli (see top row of Fig 3) as well as the exponents, $\hat{\alpha }$, derived from the slopes by the equation $\hat{\alpha }=-\frac{20}{s}\text{lg}\left(2\right)$. Note that the thresholds for the shortest duration in the 16Hz_3C condition, i.e., for MB1, deviate from a straight line. If this data point is excluded from the fit to the data, the slope is about the same for the three conditions (last row in Table 3). A slope of about -1 dB per doubling of the duration is considerably less than that expected from the exponent 3, as suggested for temporal integration in the classical audio-frequency range [25, 32, 41]; hence, the exponents that were estimated directly from the slope ($\hat{\alpha }$), are considerably higher than 3. The mismatch between these estimated exponents and the exponent that was used within the model to fit the data of the present study (i.e., 2.7) implies that the spontaneous rate (sensory noise) already substantially affects the predicted thresholds (at least for the range of durations that were considered in the present study), i.e., equation (8) of S1 File cannot be used to estimate the exponent.

**Table 3:** Parameters of the best straight-line fitted to the thresholds of the MB stimuli.

Condition	Envelope type	Slope *s* / dB	Offset *L*_O_ / dB	Exponent $\hat{\alpha }$
8Hz_3C	MB1–MB4	-1.0	106.9	6.1
16Hz_6C	MB1–MB4	-1.3	92.2	4.8
16Hz_3C	MB1–MB4	-2.3	92.2	2.7
16Hz_3C	MB2–MB4	-1.1	92.0	5.7

In the last row, the threshold for the shortest duration in the 16Hz_3C condition, i.e., for MB1, was excluded from the fit, as it deviated from a straight line.

### Plateau bursts

**Fig 5:**
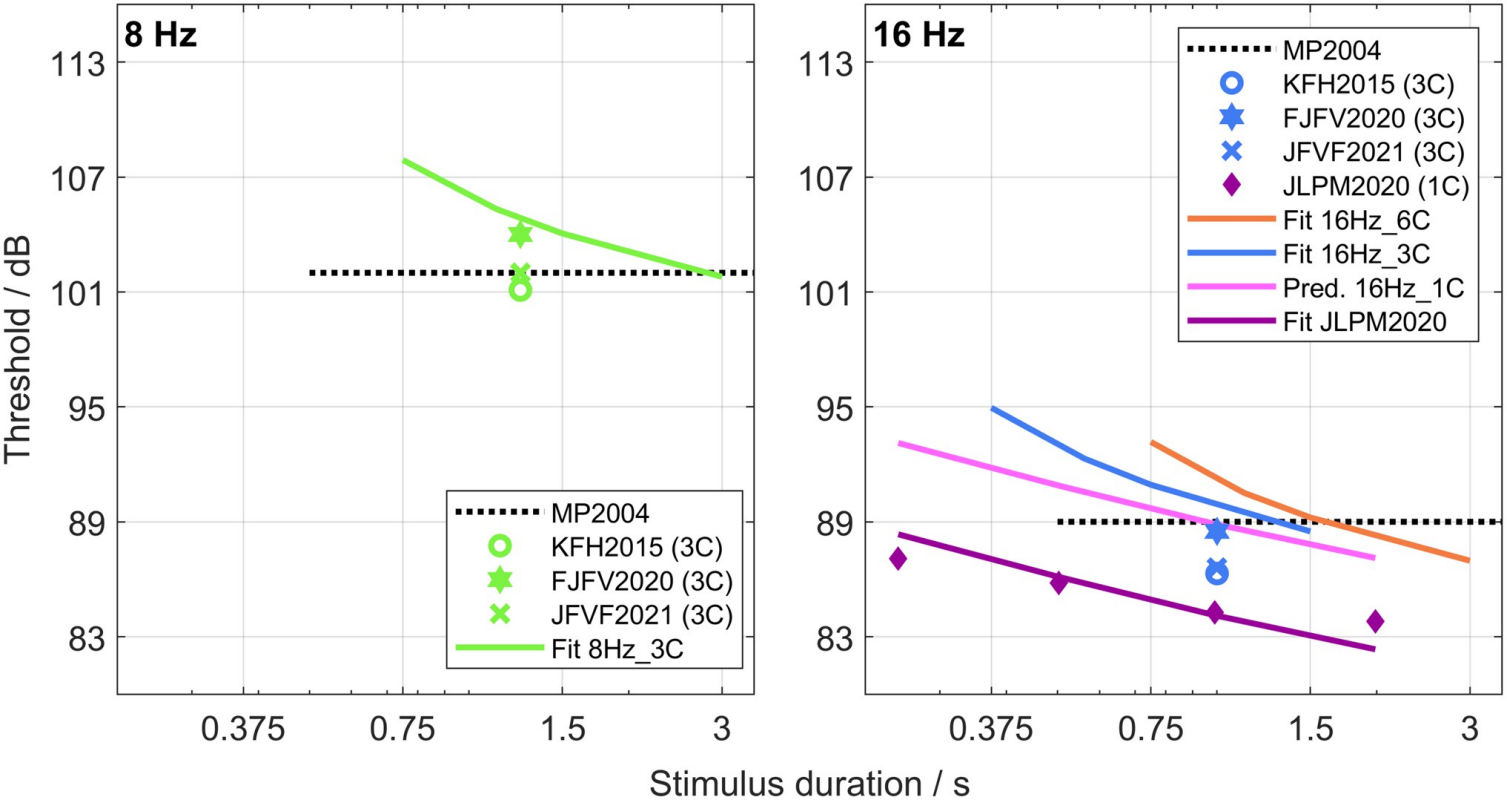
Comparison to literature data for 8 Hz (left) and 16 Hz (right) data. MP2004: Indicated as a black dotted line are the thresholds of 8 Hz and 16 Hz stimuli of various attributes published in the review by Møller and Pedersen [21]. KFH2015: The colored unfilled circles indicate the thresholds of 3C-PB stimuli reported by Kühler, Fedtke, and Hensel [17]. JFVF2021 and FJFV2020: The colored filled stars and the colored crosses represent thresholds of 3C-PB stimuli reported by Joost, Friedrich, Verhey, and Fedtke [18, 19], respectively. JLPM2020: Dark purple filled diamonds represent thresholds of 1C-PB stimuli as a function of stimulus duration reported by Jurado, Larrea, Patel, and Marquardt [20]. Light green, light red, and light blue solid lines are the fitted threshold–duration functions of PB stimuli in the three experimental conditions reproduced from Fig 4. The light purple solid line is a predicted threshold–duration function for a 16Hz_1C condition. The dark purple solid line represents the fit to the data points of JLPM2020. See text for details.

Studies on thresholds in quiet of infrasound stimuli commonly used plateau bursts instead of multiple bursts. To compare the results of the present study to the literature data, the fitted threshold–duration functions of Fig 4 for the PB stimuli in the 8Hz_3C (light green solid line in the left panel), the 16Hz_6C (light red solid line in the right panel), and the 16Hz_3C (light blue solid line in the right panel) conditions are redrawn in Fig 5. In addition, 8 Hz and 16 Hz data from the literature are shown: (a) Kühler, Fedtke, and Hensel ([17]; 3C-PB stimuli; colored unfilled circles; label KFH2015); (b) Friedrich, Joost, Fedtke, and Verhey ([19]; 3C-PB stimuli; colored filled stars; label FJFV2020); (c) Joost, Friedrich, Verhey, and Fedtke ([18]; 3C-PB stimuli; colored crosses; label JFVF2021); (d) Jurado, Larrea, Patel, and Marquardt ([20]; 1C-PB stimuli; dark purple filled diamonds; label JLPM2020). The corresponding thresholds of Møller and Pedersen ([21]; various stimulus attributes; label MP2004) are shown with horizontal black dotted lines, because duration and envelope are not well defined for these average thresholds. Note also that these latter thresholds may include a binaural advantage as they are based on several free-field and pressure-field measurements. Apart from this limitation in the comparability of the two data sets, the average thresholds of the present study are similar to the thresholds reported by Møller and Pedersen [21]. The thresholds published by Jurado, Larrea, Patel, and Marquardt [20] are shown using the same definition of duration as used in the present study. This meant adding the duration of one cycle (i.e., their ramp duration) of the infrasound sinusoid to the duration given in their study. Note that their data are considerably lower than those of the present study.

To our knowledge, the study of Jurado, Larrea, Patel, and Marquardt is currently the only study showing threshold–duration functions for infrasound. Their threshold–duration function is shallower than those of the present study. It is possible that the shallower slope is partly due to different stimulus parameters that were used in the two studies. Jurado, Larrea, Patel, and Marquardt used ramps that were only one cycle long (i.e., 62.5 ms for 16 Hz), whereas the present study used three or six cycles (i.e., 187.5 ms or 0.375 ms for 16 Hz). According to the model simulations (see bottom right panel of Fig 1 in S1 File), the threshold–duration function of 1C-PB stimuli is flatter than those for longer ramp durations. It may also be partly due to a different procedure: they used a 2-AFC procedure with three-up one-down rule, which has been generally shown to result in thresholds that differ from thresholds obtained with the three-alternative forced-choice procedure with the two-up one-down rule used in the present study [25, 41, 42].

To examine more closely how much the difference in cycle number per ramp and the different measurement procedures may have contributed to the observed deviation, the model was used to predict the threshold–duration function for a potential 16Hz_1C condition. The model parameters for this prediction were derived from the 16Hz_3C and 16Hz_6C data and by adjusting the parameters in equation (5) of S1 File to account for the two-alternative forced-choice procedure with three-up one-down rule. The resulting threshold–duration function of this prediction is shown in the right panel of Fig 5 as a light purple solid line. The predicted thresholds were higher than those measured by Jurado, Larrea, Patel, and Marquardt. To quantify the difference, the thresholds obtained by those authors were again fitted with the model such that now only the attenuation *A* was a free parameter. The threshold–duration function of this fit is shown in the right panel of Fig 5 as a dark purple solid line. It is slightly steeper than the experimental threshold–duration function, but the shape is reasonably similar. Finally, the deviation of this fit to the prediction was computed as the difference between the corresponding attenuations:

$$A\left(\text{Fit}\;\text{JLPM}2020\right)-A\left(\text{Pred.}\;16\text{Hz}\_1\text{C}\right)=83.7\;dB-88.5\;dB=-4.8\;dB$$ (4)

This means that, when the difference in the numbers of cycles per ramp as well as the measurement procedures are considered, a deviation of 4.8 dB between the data from Jurado, Larrea, Patel, and Marquardt and data of the present study is still observed. It may be that the short ramp duration results in a spectral splatter, i.e., among others, results in higher frequencies at on- and offsets. These higher frequencies may even excite filters with higher center frequencies than the lowest auditory filter, as assumed in the model to process the infrasound. This spectral splatter may also affect the slope of the threshold curve, since the ramps have a higher impact on the results at short overall durations than at long overall durations, where the plateau is much longer than the ramps. Because the input of the model is the envelope of a stimulus, it is not sensitive to spectral splatter and thus this hypothesis cannot be tested within the model framework. Note that it is equally possible that other differences, such as different cohorts of listeners or different sound-reproduction systems are mainly responsible for this discrepancy in overall threshold level. The results of the other comparable studies [17–19] are in-between the results of the present study and the results of Jurado, Larrea, Patel, and Marquardt [20]. This seems to support the hypothesis that individual differences may be the main factor to explain the difference between the various studies. Given that the numbers of participants of all these studies (except for [21]) are small, individual differences may well affect even the grand average results.

### How to define a threshold in quiet for infrasound stimuli?

An obvious choice for a definition of threshold that is based on an asymptotic behavior of the threshold–duration function would be a threshold value towards which the function converges. In the classical audio-frequency range, this value should be achieved for durations longer than about 200 ms (Fig 4.18 in [27]; however, see [32]). As mentioned in the introduction, durations of a few hundred milliseconds are too short for sinusoidal infrasound stimuli with their long periods. The data of the present study show that thresholds continue to decrease at least up to a duration of three seconds for the two frequencies that were considered. This stimulus duration is considerably longer than durations that are typically used for stimuli in the classical audio-frequency range (e.g., [22–25]).

**Fig 6:**
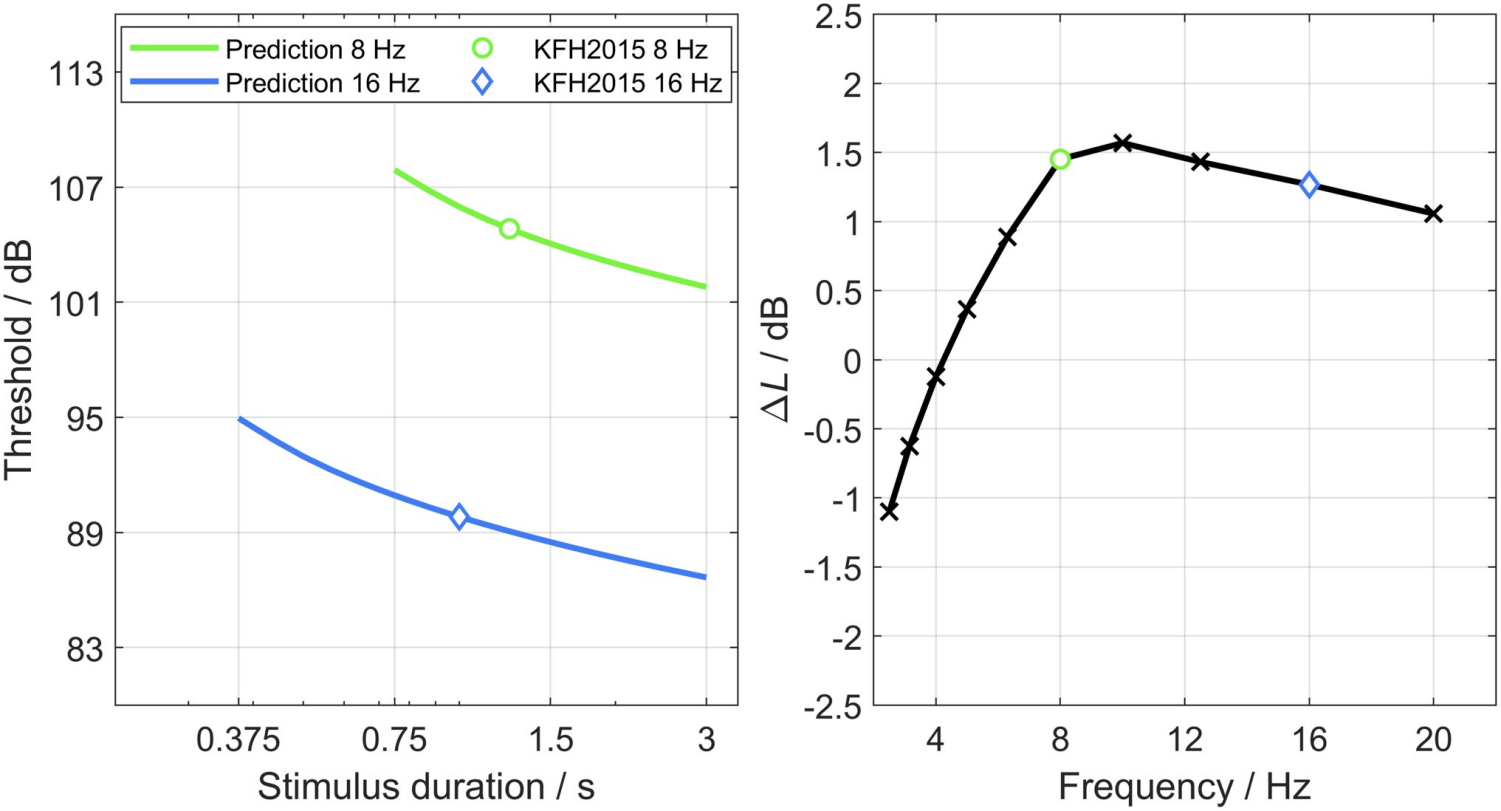
Predictions of the threshold differences relative to stimulus specifications by Kühler, Fedtke, and Hensel (KFH2015) [17]. Left: Thresholds predicted for 8 Hz and 16 Hz PB stimuli with ramp durations as specified in [17] but with varying plateau durations. For the predictions, the model with the parameters of the best fits to our data was used (see Table 2). Right: Threshold differences, Δ*L* in dB, predicted for the PB stimuli specified in [17] for frequencies from 2.5 Hz to 20 Hz. Values are highlighted for 8 Hz (green circle) and 16 Hz (blue diamond). See text for details.

To estimate thresholds for infrasound stimuli, Kühler, Fedtke, and Hensel [17] proposed frequency-dependent durations of the ramps and the plateaus of PB stimuli. For example, according to their Table 1, an 8 Hz stimulus should have three cycles per ramp and four cycles per plateau (resulting in a stimulus duration of 1250 ms) and a 16 Hz stimulus should have three cycles per ramp and ten cycles per plateau (resulting in a stimulus duration of 1000 ms). The left panel of Fig 6 shows the threshold–duration functions for 8 Hz and 16 Hz PB stimuli, predicted using the model. The thresholds for the envelopes specified in [17] are indicated by open symbols. Thus, according to the model, those thresholds do not only differ due to a different sensitivity to the two frequencies (i.e., a difference in attenuation) but also because different durations were used for the two frequencies.

To examine, how different durations affect thresholds within the model, thresholds shifts are predicted for each of the combinations of ramp duration and plateau duration recommended in [17]. The results are shown in the right panel of Fig 6. The abscissa indicates the frequency for which the combination was recommended and the ordinate the threshold shift Δ*L* in dB which is solely due to the frequency-specific envelopes (i.e., for the simulations of all frequencies, the same attenuation of *A* = 0 dB was used). For 10 Hz, a plateau duration of five cycles was used for the simulations, because this is consistent with the duration specifications in milliseconds in the last column of Table 1 in [17].

For frequencies below 8 Hz, threshold shifts are monotonically related to the frequency, since the recommended durations are equal when expressed in number of cycles, whereas the model is sensitive to the duration in milliseconds. Towards higher frequencies, the ramp and plateau durations differ between the frequencies, resulting in a slight decrease with increasing frequency. For 8 Hz (green circle) and 16 Hz (blue diamond), the predicted threshold shifts are about the same. However, if compared to, e.g., 2.5 Hz, just the choice of frequency-dependent envelopes results in a threshold difference of more than 2 dB.

The predictions of the model also challenge the thresholds proposed by Møller and Pedersen ([21]; various stimulus attributes), as these are based on stimuli with various attributes, including a wide range of stimulus durations from 0.5 s to more than 3 s. In the light of the data and model predictions of the present study, it seems difficult to define a unique threshold for a specific frequency.

An additional confounding factor is the form of the envelope. In agreement with previous studies in the classical audio-frequency range [24, 25, 32], thresholds for infrasound stimuli measured in this study were lower for PB than for MB stimuli. This finding has not yet been considered before in the definition of threshold in the infrasound frequency range. Based on the findings of the present study it seems to be necessary to include requirements of the exact envelope for each infrasound frequency in any specification of thresholds of sinusoidal infrasounds, such as Table 1 of Kühler, Fedtke, and Hensel [17]. However, as shown in Fig 6, the choice may be somewhat arbitrary.

Given these complications in estimating a threshold, it may be advantageous to find a different approach for the definition of a threshold for infrasound stimuli. A possible solution within the model framework is to use the attenuation parameter *A*. The frequency-specific values of this parameter obtained by fitting the model to the data of the present study were reasonably similar to the existing threshold data (see Table 2). Note that this attenuation can be directly derived for all data in the literature where the envelope and the psychoacoustic procedure used to estimate thresholds are well defined, if it is assumed that the spontaneous activity and the exponent are the same as used in the modelling of the present data. Under this latter constraint it is also possible to define a stimulus envelope that would be required to measure a sound pressure level at threshold that is equal to *A* (see section 4 of S1 File). In the extreme case of a rectangular envelope, this would be, for instance, a duration of *d* = 1.14 s (equation (11) of S1 File). Obviously, rectangular envelopes cannot be used due to spectral splatter at on- and offset. In general, spectral splatter can be minimized with onset and offset ramps that must be the longer the lower the frequency, such as suggested by Kühler, Fedtke, and Hensel [17]. A more complex model than the one used in the present study, i.e., one also accounting for frequency selectivity of the auditory system that is relevant for infrasound processing, is necessary to decide which is the required minimum duration of a ramp. However, the exact mechanisms underlying spectral processing of infrasound are not yet fully understood.

## Conclusions

Thresholds for the infrasound frequencies considered here strongly depend on the envelope type. The threshold–duration functions continue to decrease for durations of up to three seconds, questioning the assumption that there is a well-defined asymptotic threshold for each frequency. Thresholds of the infrasound stimuli with various envelope types were reasonably well described by the model that was originally developed to account for temporal integration in the classical audio-frequency range. Apart from the attenuation parameter, which was considerably higher than reported for the classical audio-frequency range, the parameters of the model were comparable to those used in the classical audio-frequency range. This supports the hypothesis that infrasound and sounds in the classical audio-frequency range are not processed differently by the auditory system. The thresholds of the two infrasound frequencies here were predicted by only varying the attenuation parameter of the model, assuming that the same auditory filter processes these infrasounds. This attenuation parameter offers the possibility to define a threshold that is independent of the exact duration and shape of the envelope.

## Supporting information

S1 Data Pseudonymized dataset. Contains for all listeners and experimental conditions the measured detection thresholds.
(CSV)

S1 File Model description. Describes the structure and the behavior of the model of temporal integration.
(PDF)
