# Supplementary material for: Temporal integration of infrasound at threshold
Source: PLoS One. 2023 Jul 31;18(7):e0289216. doi: 10.1371/journal.pone.0289216 (PMC10389702; doi:10.1371/journal.pone.0289216)
Supplement: S1 File — Describes the structure and the behavior of the model of temporal integration. (PDF) [file pone.0289216.s002.pdf]

Contents

1 **Model structure** 1

2 **Model behavior** 2

3 **Predictions for MB stimuli, assuming $\lambda_{\text{spont}} = {}^0\!/\!_\text{s}$** 4

4 **Duration of a rectangular envelope required for $L = A$** 5

1 Model structure

The model of temporal integration described here is a modified version of the physiologically inspired probabilistic model proposed by Heil, Matysiak, and Neubauer [25]. The auditory stimuli are represented by "event" rates, which can be thought of as generalized neuronal firing rates. These are modeled by an inhomogeneous Poisson process, which is often assumed to describe neuronal activity (e.g., [40]). The time-dependent rate of the events is denoted as $\lambda(t)$ and has the unit reciprocal second. It is the sum of a spontaneous event rate, λ_{spont}, and a stimulus-driven time-varying rate. In the original model, this rate is proportional to the time-varying stimulus envelope, $M \cdot f(t)$, raised to the power of an exponent α, i.e.,

$$\lambda(t) = \lambda_{\text{spont}} + g \cdot \left[M \cdot f(t)\right]^{\alpha} \; . \tag{1}$$

Here, M denotes the maximum stimulus amplitude and $f(t)$ represents the normalized, time-dependent envelope after peripheral filtering (i.e., applying a gammatone filter). The proportionality factor g reflects the sum of all gains within the auditory system as a linear measure and has units of $\text{Pa}^{-\alpha}{\cdot}\text{s}^{-1}$.

The following modifications of the original model described in [25] were necessary: Thresholds of infrasound stimuli may well exceed sound pressure levels of 100 dB. Because the gain factor g and the exponent α interact, undesirable numerical effects may occur when fitting this model to detection thresholds at these high sound pressure levels. Therefore, instead of the gain factor, an attenuation A was used, which is related to g via the relationship

$$A = -\frac{20}{\alpha} \lg\left(g\right) \text{dB} \, . \tag{2}$$

Another modification concerns the peripheral filter. In the modified model, stimulus envelopes were not filtered, since the properties of such a hypothetical filter for infrasound stimuli are unclear. With these modifications, the rate equation (1) in this supplement becomes:

$$\lambda(t) = \lambda_{\text{spont}} + 10^{-\alpha \cdot A/20\,\text{dB}} \cdot \left[M \cdot f(t)\right]^{\alpha} \; . \tag{3}$$

To obtain a threshold, the event rate $\lambda(t)$ is, as in the original version, integrated over an evaluation interval d. This evaluation interval starts with the onset of each interval of a trial in the AFC procedure and has the same duration as the interval. In our study, the duration of the evaluation interval was always identical to the stimulus duration. Within the model, the signal is detected, if

$$\int_0^d \lambda(t)\,\text{d}t \geq n_{\text{crit}} \, , \tag{4}$$

where n_{crit} is the critical mean number of events during the stimulus interval. The critical mean number of events depends on the number of spontaneous events, $n_{\text{spont}} = \lambda_{\text{spont}} \cdot d$, on the number of intervals, n_{alt}, and on the targeted probability of correct decisions, p_{corr}. The relationship can be described by

$$n_{\text{crit}} = \left(n_{\text{crit}}^{a_0}(0) + \sum_{i=0}^{3} b_i n_{\text{spont}}^{a_i} \right)^{1/a_0} \qquad \text{with} \quad n_{\text{crit}}(0) = \frac{1 - 1/n_{\text{alt}}}{1 - p_{\text{corr}}} \,. \tag{5}$$

The coefficients of this function, and hence its shape, depend on the specific measurement procedure (for details see [25, 41, 42]). Table 1 in this supplement summarizes the values of the coefficients for different measurement procedures. For example, for the adaptive 3-AFC procedure with a one-up two-down rule (i.e., for $n_{\text{alt}} = 3$ and $p_{\text{corr}} \approx 0.707$), which was used in the present study, the values of the first row are used.

Table 1: Coefficients used to define n_{crit} in equation (5) in this supplement. The values were reproduced from those in Table A1 of [42].

n_{alt}	p_{corr}	a_0	a_1	a_2	a_3	b_0	b_1	b_2	b_3
3	0.707	1.8091	0.9728	1.1015	1.3533	1	2.6579	0.0138	1.5240
3	0.794	3.1736	0.6091	1.5980	2.6081	1	2.7523	31.805	8.5107
2	0.707	5.1091	1.5926	3.4925	4.5452	1	3.6440	21.186	6.2874
2	0.794	1.9311	1.0513	1.0577	1.4962	1	2.8143	0.0028	1.4034

The detection threshold is calculated from the critical maximum amplitude, M_{crit}, for which the critical number of events is reached or exceeded:

$$M_{\text{crit}}^{\alpha} = \frac{n_{\text{crit}} - n_{\text{spont}}}{\int_0^d f^{\alpha}(t)\,\mathrm{d}t} \cdot 10^{\alpha \cdot A/20\,\text{dB}} \,. \tag{6}$$

Expressed in terms of decibels, the detection threshold is given by

$$L = 20 \cdot \lg\left(M_{\text{crit}}\right) \text{dB} = A + \frac{20}{\alpha} \cdot \lg\left(\frac{n_{\text{crit}} - n_{\text{spont}}}{\int_0^d f^{\alpha}(t)\,\mathrm{d}t} \right) \text{dB} \,. \tag{7}$$

2 Model behavior

Heil, Matysiak, and Neubauer [25] showed that the parameters of their model (bandwidth of the peripheral filter, exponent, and spontaneous rate) affect the predictions of the model (see Fig. 10 in [25]). To understand how changes in the parameters α, A, and λ_{spont} of the model used in the present study (with $n_{\text{alt}} = 3$ and $p_{\text{corr}} \approx 0.707$; cf. Table 1) affect the predicted threshold–duration functions, simulations were conducted in which one parameter was systematically varied while the other two were held constant. Test stimuli were MB and PB stimuli with a frequency of 16 Hz — similar to those used in the experiments but with broader ranges of stimulus duration (0.125 s to 7.5 s). Because the ramp durations used in the literature and in the experiments of the present study differ, it was additionally investigated how various ramp durations ranging from 62.5 ms (i.e., one cycle re 16 Hz) to 375 ms (i.e., six cycles re 16 Hz) affect the results. Figure 1 in this supplement shows, for a selected set of these MB (left column) and PB (right column) stimuli, how predicted

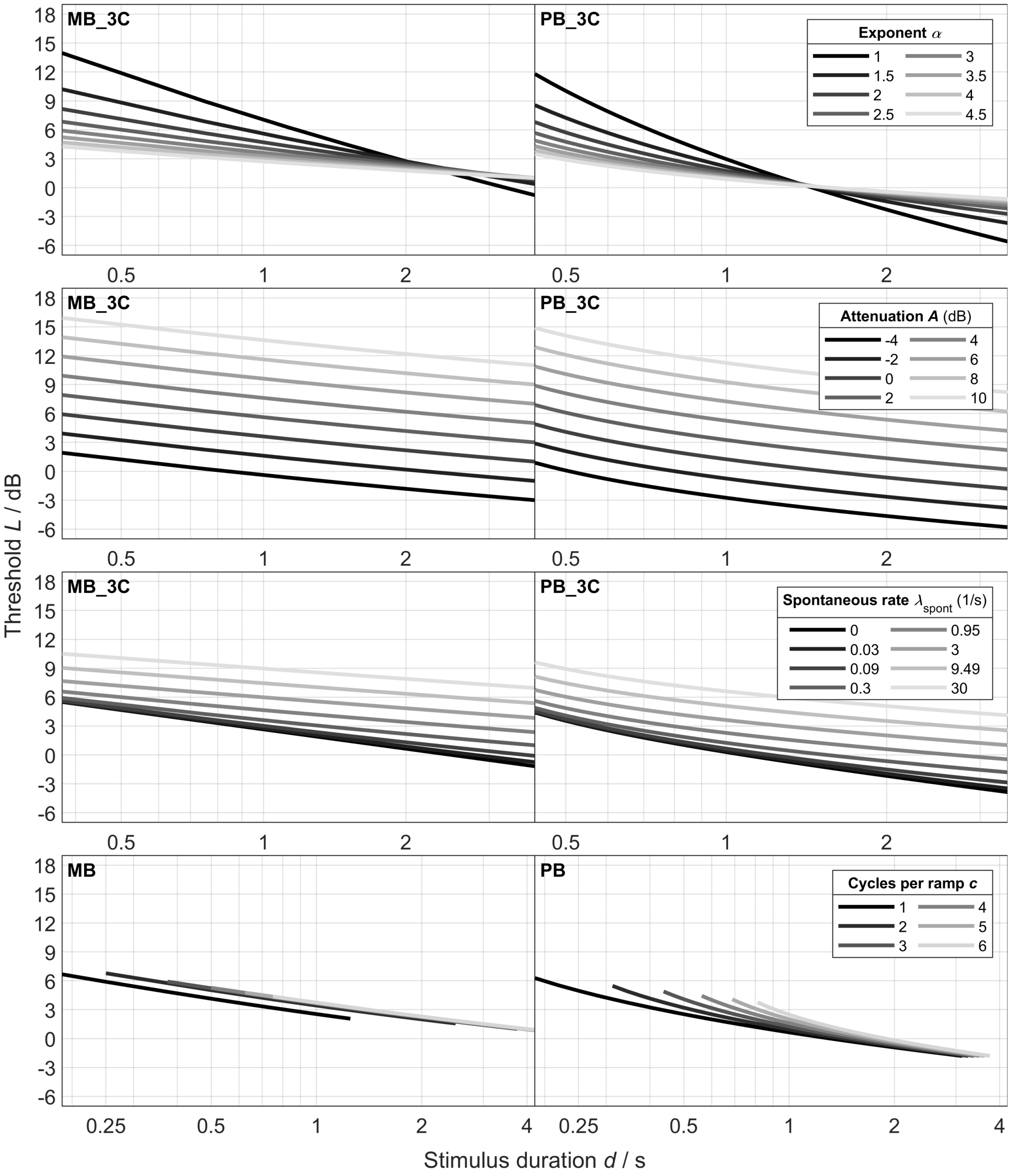

Figure 1: Effects of varying the model parameters and ramp durations on threshold–duration functions for MB (left panels) and PB (right panels) stimuli. Top row of panels: The exponent α determines the general slope of the threshold–duration function. The larger α, the flatter the slope of the function. Second row of panels: The attenuation A shifts the entire function relative to a baseline curve with $A = 0\,\mathrm{dB}$. Third row of panels: The spontaneous rate λ_{spont} affects the curvature of the function. The higher the spontaneous rate, the stronger the curvature. The effect is the more pronounced the longer the stimulus duration. Bottom panels: The number of cycles per ramp c affects the shape of the function in a complex way. See text for details.

threshold–duration functions change when the model parameters (for 3C stimuli only) or ramp durations are varied.

In the top panels of Figure 1 in this supplement, the exponent α was varied from 1 to 4.5 in steps of 0.5; the spontaneous rate and the attenuation were set to $\lambda_{\text{spont}} = {}^{0.3}/\text{s}$ and $A = 0\,\text{dB}$, respectively. For both envelope types, an increase in the exponent leads to a decrease in the slope of the threshold–duration function. The difference between the threshold–duration functions for different values of the exponent is largest when stimuli are very short or very long.

In the second row of panels of Figure 1 in this supplement, A was varied from -4 dB to 10 dB in steps of 2 dB; the exponent was set to $\alpha = 3$ and the spontaneous rate to $\lambda_{\text{spont}} = {}^{0.3}/\text{s}$. For both envelope types, changing A results in a vertical shift of the threshold–duration functions. The vertical distance of two threshold–duration functions with different values for A is equal to the difference between the two A values. This is true regardless of the values of the other parameters as long as they are the same for the two threshold–duration functions that are compared (cf. equation (7) in this supplement). This further motivates the choice of the parameter A, since it is easier to interpret than the gain factor g of the original model (the latter implicitly depends on α).

In the third row of panels of Figure 1 in this supplement, the spontaneous rate λ_{spont} was varied from ${}^{0.3}/\text{s}$ to ${}^{30}/\text{s}$ in multiplicative steps of $\sqrt{10}$. In addition, predictions with $\lambda_{\text{spont}} = {}^{0}/\text{s}$ are shown. For all predictions shown in these bottom panels, the exponent was $\alpha = 3$ and the attenuation $A = 0\,\text{dB}$. For this latter extreme case, the model predicts, for the MB stimuli, a linear relation between threshold and the logarithm of the number of bursts (and, hence, between threshold and the logarithm of stimulus duration). A non-zero spontaneous rate introduces a curvature into the threshold–duration functions, mostly affecting thresholds at the longest stimulus durations. Even though this is true for the whole range of non-zero spontaneous rates considered here, this curvature is best seen when spontaneous rates are low (here up to about ${}^{0.95}/\text{s}$). At higher spontaneous rates, threshold–duration functions appear to be shifted as a whole, because then the effect of spontaneous rate is observed for all durations that are considered in the simulations.

In the bottom panels of Figure 1 in this supplement, the ramp duration (expressed as the number of cycles per ramp c) was varied from 1 to 6 in steps of 1. The other parameters of the model were set to $\alpha = 3$, $A = 0\,\text{dB}$, and $\lambda_{\text{spont}} = {}^{0.2}/\text{s}$. The ramp duration affects the shape of threshold–duration functions in a more complex way than the three model parameters. For the MB stimuli, differences in threshold between consecutive functions decrease with increasing c; the largest threshold difference (≈ 1 dB) is observed between the functions for $c = 1$ and $c = 2$. The effect of c is different for PB stimuli: The slope of the functions increases as c increases. All functions converge towards longer stimulus durations.

3 Predictions for MB stimuli, assuming $\lambda_{\text{spont}} = {}^{0}/\text{s}$

MB stimuli (without gaps) have the following nice property: The integral of an MB stimulus with m bursts, $J(m)$, is m times the integral of the MB stimulus with only one burst, $J(1) = \int_0^d f^\alpha \,\text{d}t$. Exploiting this property, equation (7) in this supplement for the prediction of the threshold can be simplified under the assumption that $\lambda_{\text{spont}} = {}^{0}/\text{s}$. In this case, $n_{\text{crit}} - n_{\text{spont}} = n_{\text{crit}}(0)$, which is a constant specific for the measurement procedure, and equation (7) in this supplement can be re-written as a function of m as follows:

$$
\begin{aligned}
L(m) &= A + \frac{20}{\alpha} \cdot \lg\left(\frac{n_{\text{crit}} - n_{\text{spont}}}{J(m)}\right) \text{dB} \\
&= A + \frac{20}{\alpha} \cdot \lg\left(\frac{n_{\text{crit}}(0)}{m \cdot J(1)}\right) \text{dB} \\
&= L(1) - \frac{20}{\alpha} \cdot \lg(m)\, \text{dB}\,.
\end{aligned}
\tag{8}
$$

In words: If there is no spontaneous rate, the threshold function is linear when plotted as a function of lg(m), with the slope being $-20/\alpha$ and the offset being the threshold of MB1.

4 Duration of a rectangular envelope required for $L = A$

It may be of interest to know the duration d that a stimulus must have in order for the sound pressure level at threshold L to be equal to A. From equation (7) in this supplement it follows that this is so if

$$\int_0^d f^{\alpha}(t)\, \mathrm{d}t = n_{\text{crit}} - n_{\text{spont}}\,. \tag{9}$$

In the case of a sinusoidal stimulus without ramps, the envelope is a rectangle, which simplifies equation (9) in this supplement to

$$d/\text{s} = n_{\text{crit}} - n_{\text{spont}}\,. \tag{10}$$

With $n_{\text{spont}} = \lambda_{\text{spont}} \cdot d$, this can be equivalently transformed to

$$d = \frac{n_{\text{crit}}}{1/\text{s} + \lambda_{\text{spont}}}\,. \tag{11}$$

In words: In order for the threshold L to be equal to the attenuation A of a rectangular sinusoidal stimulus, the duration d is solely determined by the spontaneous rate λ_{spont} and the critical number of spikes n_{crit}. This duration does not depend on the exponent α. For example, if a 3-AFC procedure with a two-up one-down rule is used and if $\lambda_{\text{spont}} = 0/\text{s}$, then $d = 0.82$ s.
